# Protection by Salidroside against Bone Loss via Inhibition of Oxidative Stress and Bone-Resorbing Mediators

**DOI:** 10.1371/journal.pone.0057251

**Published:** 2013-02-20

**Authors:** Jin-Kang Zhang, Liu Yang, Guo-Lin Meng, Zhi Yuan, Jing Fan, Dan Li, Jian-Zong Chen, Tian-Yao Shi, Hui-Min Hu, Bo-Yuan Wei, Zhuo-Jing Luo, Jian Liu

**Affiliations:** 1 Institute of Orthopedic Surgery, Xijing Hospital, Fourth Military Medical University, Xi′an, People's Republic of China; 2 Research Center of Traditional Chinese Medicine, Xijing Hospital, Fourth Military Medical University, Xi′an, People's Republic of China; 3 Department of Pharmacology, School of Pharmacy, Fourth Military Medical University, Xi'an, People's Republic of China; The University of Hong Kong, Hong Kong

## Abstract

Oxidative stress is a pivotal pathogenic factor for bone loss in mouse model. Salidroside, a phenylpropanoid glycoside extracted from Rhodiola rosea L, exhibits potent antioxidative effects. In the present study, we used an in vitro oxidative stress model induced by hydrogen peroxide (H_2_O_2_) in MC3T3-E1 cells and a murine ovariectomized (OVX) osteoporosis model to investigate the protective effects of salidroside on bone loss and the related mechanisms. We demonstrated that salidroside caused a significant (*P*<0.05) elevation of cell survival, alkaline phosphatase (ALP) staining and activity, calcium deposition, and the transcriptional expression of *Alp*, *Col1a1* and *Osteocalcin* (*Ocn*) in the presence of H_2_O_2_. Moreover, salidroside decreased the production of intracellular reactive oxygen species (ROS), and osteoclast differentiation inducing factors such as receptor activator of nuclear factor-kB ligand (RANKL) and IL-6 induced by H_2_O_2_. In vivo studies further demonstrated that salidroside supplementation for 3 months caused a decrease in malondialdehyde (MDA) and an increase in reduced glutathione (GSH) concentration in blood of ovariectomized mouse (*P*<0.05), it also improved trabecular bone microarchitecture and bone mineral density in the fourth lumbar vertebra and distal femur. Our study indicated that the protection provided by salidroside in alleviating bone loss was mediated, at least in part, via inhibition of the release of bone-resorbing mediators and oxidative damage to bone-forming cells, suggesting that salidroside can be used as an effective remedy in the treatment or prevention of osteoporosis.

## Introduction

Osteoporosis is a degenerative bone disease characterized by low bone mass and structural deterioration of bone tissue, leading to bone fragility [1]. Oxidative stress, resulting from excessive generation of reactive oxygen species, can damage all components of the cell. At present, numerous studies have shown the positive correlation between oxidative stress and osteoporotic status. For instance, in osteoporotic postmenopausal women, decreased bone mineral density was shown to be associated with higher plasma lipid oxidation [2], and catalase and glutathione peroxidase activity were found to be lowered [3], [4]. Ovariectomy induces oxidative stress in rat femurs together with a decreased activity of antioxidant systems [5]. Evidence mainly obtained from the studies in mouse model provides a paradigm shift from the ‘estrogen-centric’ account of the pathogenesis of involutional osteoporosis to one in which age-related mechanisms intrinsic to bone and oxidative stress are protagonists, and moreover, age-related changes in other organs and tissues, such as ovaries, accentuate them [6].

Bone mass is maintained through a dynamic balance between osteoblastic bone formation and osteoclastic bone resorption [7]. Evolving evidence suggests that reactive oxygen species can enhance bone resorption by directly or indirectly promoting osteoclasts formation and activity [8]. However, it can induce apoptosis of osteoblasts and decrease their activity leading to reduced osteoblastic bone formation [9]. As a result, the imbalance between these two types of cell leads to bone metabolic diseases, such as osteoporosis. Postmenopausal osteoporosis is associated with significant changes in bone turnover: bone formation decreases and bone resorption increases or remains unchanged, leading to a net bone loss [10]. Therefore, therapeutic strategies aimed at preventing or delaying reactive oxygen species might be a reasonable choice for the treatment of osteoporosis. Recently, attention has been focused on searching for natural substances with antioxidative potential that can scavenge free radicals and protect cells from oxidative damage.

**Figure 1:**
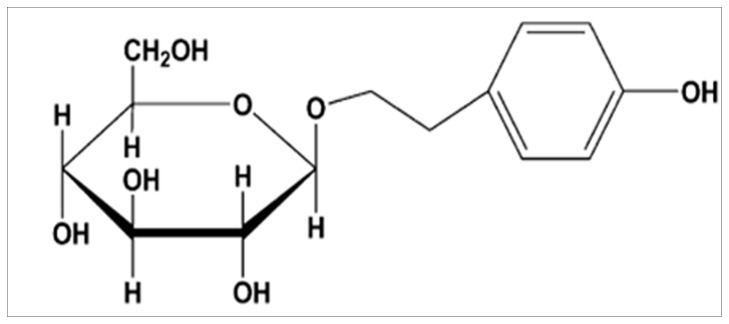
Chemical structure of salidroside.

Salidroside (structure shown in Fig. 1) is an active constituent extracted from the root of Rhodiola rosea L, which has been used as a medicinal herb for a long time. Reports have existed in the literature that salidroside possesses anti-aging, anti-cancer, anti-inflammatory, anti-hypoxia and antioxidative properties [11], [12], [13], [14]. Moreover, recent studies have shown that salidroside protects human erythrocytes against hydrogen peroxide (H_2_O_2_) -induced apoptosis and protects against H_2_O_2_-induced injury in cardiac H9c2 cells via PI3K-Akt dependent pathway [15], [16]. Our previous studies revealed that salidroside protects against 1-methyl-4-phenylpyridinium (MPP+)-induced apoptosis in PC12 cells by inhibiting the NO pathway, it can also provide neuroprotective effects against focal cerebral ischemia in vivo and H_2_O_2_-induced neurotoxicity in vitro [17], [18].

However, whether salidroside can provide protection against bone loss associated with oxidative stress remains unknown. Therefore, in this study, we used an in vitro oxidative stress model induced by H_2_O_2_ in MC3T3-E1 cells, which are preosteoblastic cells from mouse calvariae commonly used in studying osteogenic development [19], and an in vivo ovariectomized (OVX) osteoporosis model in mouse to investigate the protective effects of salidroside on bone loss as well as the underlying mechanisms.

## Materials and Methods

### Materials

Salidroside extracted from Rhodiola rosea L (purity>99%) was obtained from National Institute for the Control of Pharmaceutical and Biological Products (Beijing, China). Fetal bovine serum (FBS) and α-Modified minimal essential medium (α-MEM) were purchased from Gibco Life Technologies (Grand Island, USA). ALP activity assay kit was purchased from GENMED scientific Inc. (USA). RANKL and IL-6 ELISA kit were purchased from R&D system Inc. (Minneapolis, MN, USA). PrimeScript RT reagent kit and SYBR Premix Ex Taq were obtained from TaKaRa Biotechnology (Dalian, China). Oligonucleotide primers were synthesized by Sangon Biological Engineering Technology Co. (Shanghai, China). BCIP/NBT Alkaline Phosphatase Color Development Kit,RIPA Lysis Buffer, Reactive Oxygen Species Assay Kit, GSH and MDA Assay kit were purchased from the Beyotime Institute of Biotechnology (Shanghai, China). All other chemicals and reagents were of analytical grade.

### Cell culture

Murine osteoblastic MC3T3-E1 cells were obtained from the Center Laboratory for Tissue Engineering, College of Stomatology, Fourth Military Medical University, Xi’an, China [20], [21]. MC3T3-E1 cells were maintained in a-MEM with 10% heat-inactivated FBS and 100 U/ml penicillin and 100µg/ml streptomycin under conditions of 5% CO_2_ and 37°C. H_2_O_2_ was used as the exogenous ROS source, and N-acetyl-L-cysteine (NAC) was used as an ROS scavenger. When reaching confluence, MC3T3-E1 cells were treated with culture medium containing 10 mM β-glycerophosphate and 50µg/ml ascorbic acid to initiate differentiation. After 6 or 14 days, the cells were pre-incubated with serum-free regular culture medium containing salidroside for 24 h before treatment with 300µM H_2_O_2_ for another 24 h. In all the experiments, salidroside treatment continued after the pretreatment. Each experiment was performed in duplicate wells and repeated three times.

### Assays of cell viability

In the experiments, cells were treated with different concentrations of H_2_O_2_ (0, 100, 200, 300, 400µM) for 2, 6, 12, 24h or salidroside (0, 0.1, 1, 10µM) for 24, 72h to investigate the toxicity of H_2_O_2_ and salidroside. Then, cells were cultured with serum-free regular culture medium containing salidroside (0, 0.1, 1, 10µM) for 24 h followed by treatment with 300µM H_2_O_2_ for 24 h. Cell viability was measured by MTT assay. In brief, 0.02 ml MTT solution was added to each well and the plates were incubated at 37 °C for 4 h. Then DMSO was added and the plates were shaken for 5 min to dissolve formazan products. The absorbance of each well was recorded on a microplate reader at a wavelength of 492 nm. Cell viability of the control group not exposed to either H_2_O_2_ or salidroside was defined as 100%.

### Alkaline phosphatase (ALP) staining and activity assay

After 6 days, the cells were cultured with serum-free medium containing salidroside and/or H_2_O_2_ for 2 days. At harvesting, cells were washed twice with phosphate-buffered saline (PBS), fixed with 10% formalin in PBS for 30 s, rinsed with deionized water, and stained with BCIP/NBT Alkaline Phosphatase Color Development Kit under protection from direct light. To measure ALP activity, the cell monolayer was lysed with RIPA. The lysate was centrifuged at 10,000×g for 5 min. The clear supernatant was used to measure the ALP activity, which was determined using an ALP activity assay kit. Total protein concentrations were determined by Bradford protein assay method. ALP activity was normalized to total protein measured with the Bradford protein assay method.

### Calcium deposition assay

After 14 days, the cells were cultured with serum-free medium containing salidroside and/or H_2_O_2_ for 2 days. At harvesting, the cells were fixed in ice-cold 10% formalin for 20 min and stained with 40 mM Alizarin Red S (pH 4.4, Sigma) for 45 min at room temperature. For matrix calcification estimate, the stain was solubilized with 10% cetylpyridinum chloride by shaking for 15 min. The absorbance of the released Alizarin red S was measured at the wave length of 562 nm [22].

### Quantitative real-time PCR

After 6 days, the cells were cultured with serum-free medium containing salidroside and/or H_2_O_2_ for 2 days. Total cellular RNA was extracted using the Trizol reagent according to the manufacturer's instructions (OMEGA). Single strand cDNA synthesis was performed by using PrimeScript RT reagent kit (TaKaRa). Real-time quantitative RT-PCR was performed using the CFX96 (BIO-RAD) instrument and individual PCRs were carried out in 96-well optical reaction plates with SYBR Green-I (TaKaRa) in accordance with the manufacturer's instructions. Briefly, PCR was carried out in a 25-µl final volume containing the following reagents: 12.5 µl of 2× SYBR Green-I master mix, 1 µl of 10 µM each primer, 2.5 µl of 1 µg/µl cDNA template, and 8 µl of double deionized water. PCR conditions were as follows: initial denaturation at 95°C for 30 s, followed by 40 cycles of denaturation at 95°C for 5 s, and annealing at 58°C for 15 s. Fluorescent product was measured by a single acquisition mode at 58°C after each cycle. Target genes (*Alp*, *Col1a1*, *Osteocalcin* (*Ocn*)) expression was normalized to the reference gene β-actin. The 2^−ΔΔCt^ method was applied to calculate relative gene expression. The PCR products were subjected to melting curve analysis and standard curve to confirm the correct amplification. All the RT-PCRs were performed in triplicates and the primers used for PCR are listed in Table 1.

**Table 1:** Real-time PCR primers for amplification of specific MC3T3-E1 mRNA.

Gene	Forward (5′–3′)	Reverse (5′–3′)
*Alp*	aacccagacacaagcattcc	ccagcaagaagaagcctttg
*Col1a1*	gcatggccaagaagacatcc	cctcgggtttccacgtctc
*Ocn*	ctgacaaagccttcatgtccaa	gcgccggagtctgttcacta
β-actin	ctggcaccacaccttctaca	ggtacgaccagaggcataca

### Measurement of RANKL and IL-6

After 6 days, the cells were cultured with serum-free medium containing salidroside and/or H_2_O_2_ for 2 days. RANKL and IL-6 contents in culture medium were measured using sandwich ELISA assay kit, according to the manufacturer's recommendation. Total protein concentrations were determined by Bradford protein assay method.

### Measurement of intracellular ROS

The level of intracellular ROS was quantified using Reactive Oxygen Species Assay Kit. DCFH-DA is oxidized by ROS in viable cells to 2′,7′-dichlorofluorescein (DCF) which is highly fluorescent at 530 nm. The cells were washed three times with PBS. DCFH-DA, diluted to a final concentration of 10 µM, was added and incubated for 30 min at 37°C in the dark. After being washed three times with PBS, the relative levels of fluorescence were quantified using a multi-detection microplate reader (485 nm excitation and 535 nm emission).

### Animals and salidroside treatments

Thirty-two 9-week-old and weighing 20.3 ±1.34 g BALB/c female mice were purchased from the Experimental Animal Center of The Fourth Military Medical University (Xi’an, China) and were acclimated to laboratory conditions for 1 week before the experiment. The initial body weight of the mice was no significant difference among the four groups in this study. They were maintained in a well-ventilated controlled room at 20°C on a 12-h light/dark cycle with free access to water and food. Mice underwent sham-operation (n = 8) or were surgically ovariectomized (OVX; n = 24) under anesthesia by pentobarbital sodium (50 mg/kg body weight, i.p.). OVX was performed by removing the bilateral ovaries through a dorsal approach and sham surgery was performed by identifying the bilateral ovaries. The mice were randomly divided into four groups: (1) untreated (Sham: sham-operated controls); (2) untreated (OVX controls); (3) OVX administered intraperitoneally with salidroside (5 mg/kg body weight) daily; (4) OVX administered intraperitoneally with salidroside (20 mg/kg body weight) daily. Salidroside were dissolved in distilled water and distilled water alone was administered to untreated mice. The treatments started 1week after the surgery and lasted for 15 weeks. Blood samples were taken from the heart in anesthetized mice and serum was then prepared by centrifugation. The left femur and the 4 th lumbar vertebra (L4) from each mouse were removed and cleaned of adherent tissue. All experimental procedures in animals were approved by the Ethics in Animal Research Committee of the Fourth Military Medical University (permission code 2010C00843).

### Measurements of serum reduced glutathione (GSH) and malondialdehyde (MDA)

The GSH in whole blood samples were determined using a GSH Assay kit following the manufacturer's instructions. Determination of GSH is based on the reaction of GSH with 5.50-dithiobis-2-nitrobenzoic acid (DTNB) to form a product that can be detected by a spectrophotometer at 412 nm. The MDA in whole blood samples were determined using a Lipid peroxidation MDA Assay Kit following the manufacturer's instructions. The binding of thiobarbituric acid to MDA which was formed during lipid peroxidation results in a chromogenic complex.

### Bone microarchitecture assessment by micro-computed tomography

Bone microarchitecture in the 4 th lumbar vertebra (L4) and distal femur was scanned using explore Locus SP Pre-Clinical Specimen micro-computed tomography (GE Healthcare, USA) with 8-µm resolution, tube voltage of 50 kV and tube current of 0.1 mA. The reconstruction and 3D quantitative analyses were performed by using software provided by a desktop micro-CT system (GE Healthcare, USA). The same settings for scan and analysis were used for all samples. In the femora, scanning regions were confined to the distal metaphysis, extending proximally 2.0 mm from the proximal tip of the primary spongiosa. Trabecular bone region from the vertebral body was outlined for each micro-CT slice, excluding both the cranial and caudal endplate regions. Within these regions, trabecular bone was separated from cortical bone with boundaries defined by the endocortical bone surfaces. The following 3D indices in the defined region of interest (ROI) were analyzed: relative bone volume over total volume (BV/TV, %), trabecular number (Tb.N), trabecular separation (Tb.Sp), trabecular thickness (Tb.Th), connectivity density (Conn.D), structure model index (SMI) and bone mineral density (BMD).The operator conducting the scan analysis was blinded to the treatments associated with the specimens.

### Undecalcified histological examination

The left femur of each mouse was collected and fixed in 4% paraformaldehyde for 48 hours. All samples were dehydrated in graded alcohols and embedded in polymethyl-methacrylate (PMMA). The distal femurs were cut into sections of 5 and 240µm in thickness in the coronal plane on a rotation microtome. The 5µm sections were stained with Von kossa staining to visualize calcium deposition and 240µm sections were hand grounded to a thickness of 20µm for Van Gieson staining, which is used to stain collagen fiber.

### Statistical analysis

Data were expressed as means ± S.D. of multiple repeats of the same experiment (n = 5). The data for these measurements were analyzed with one-way analysis of variance (ANOVA) with subsequent post hoc multiple comparison by Dunnett's test. Statistically significant values defined as *P*<0.05.

## Results

### Protective effect of salidroside on H_2_O_2_ induced cytotoxicity in MC3T3-E1 cells

**Figure 2:**
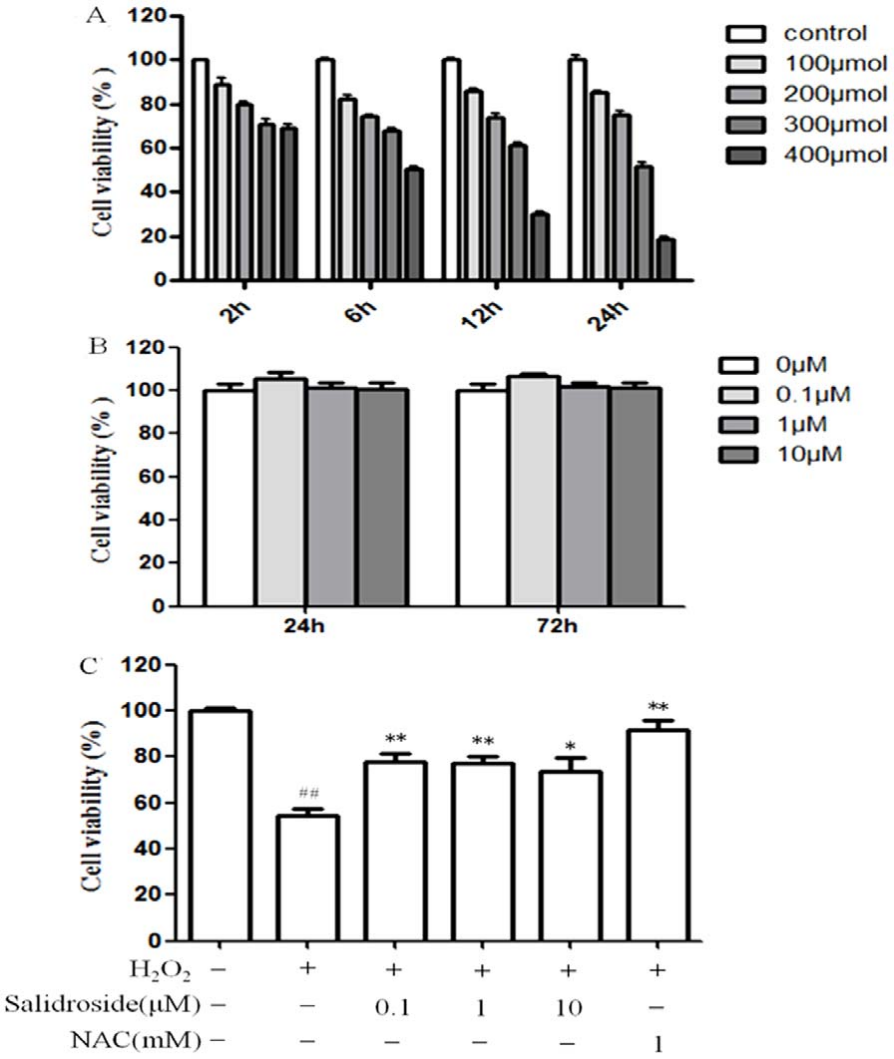
Protective effect of salidroside on H_2_O_2_ induced cytotoxicity in MC3T3-E1 cells. A: Concentration- and time-dependent effects of H_2_O_2_ on cell viability. B: Cells were incubated in different concentrations of salidroside alone. C: Cells were pretreated with salidroside for 24 h before treatment with 0.3 mM H_2_O_2_ for 24 h. NAC was used as positive control. The control value for cell viability was 0.431±0.05 OD. ^##^ *P*<0.01 versus untreated control cells; *P<0.05 and **P<0.01 compared with the group treated with H_2_O_2_ alone.

Cell viability was analyzed to determine the protective effect of salidroside on the response of MC3T3-E1 cells to oxidative stress induced by H_2_O_2_. As shown in Fig.2A, 100 to 400µM of H_2_O_2_ induced significant decrease in cell survival in a dose- and time-dependent manner. In the group treated with 300 µM H_2_O_2_ for 24 h, cell viability was significantly decreased by approximately 50% as compared with the control group(*P*<0.01). Therefore, the treatment of 300 µM H_2_O_2_ for 24 h was used to induce MC3T3-E1 cell injury in subsequent experiments. As shown in Fig. 2B, salidroside alone was non-toxic to cells at the concentrations used in this study (*P*>0.05). When the cells were pretreated with salidroside for 24 h before the addition of H_2_O_2_ (300µM) for 24 h, salidroside (0.1∼10µM) significantly increased the survival of MC3T3-E1 cells as compared to H_2_O_2_ alone treated cells (*P*<0.05), suggesting that salidroside suppressed H_2_O_2_ induced cytotoxicity (Fig. 2C). NAC, which significantly inhibited H_2_O_2_–induced cytotoxicity at 1mM as a potent antioxidant, was used here serving as a positive control.

### Protective effect of salidroside on osteoblast dysfunction induced by H_2_O_2_

**Figure 3:**
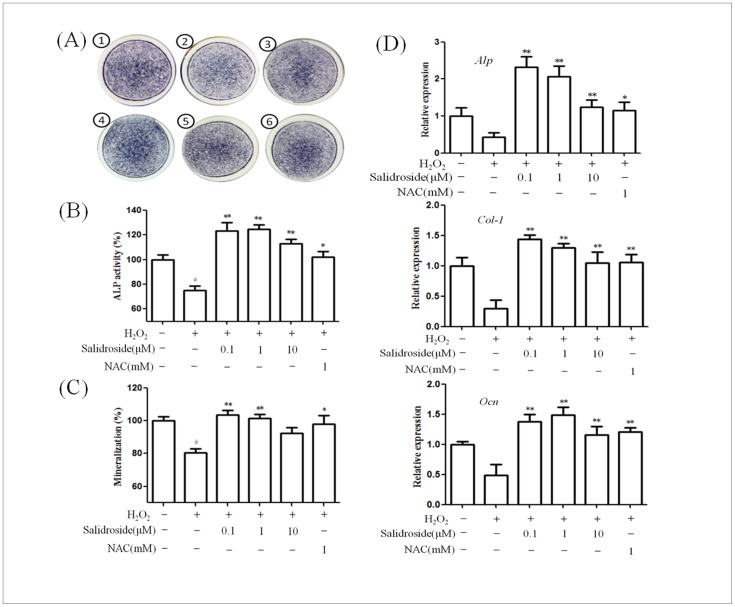
Protective effect of salidroside on H_2_O_2_ induced osteoblast dysfunction. After differentiation induction of 6 or 14 days, MC3T3-E1 cells were pretreated with salidroside for 24 h before treatment with 0.3 mM H_2_O_2_ for 24 h. NAC was used as positive control. A: Effect of salidroside on the ALP staining of MC3T3-E1 cells in the presence of H_2_O_2_. <$>\raster(70%)="rg1"<$> the control group; <$>\raster(70%)="rg2"<$> H_2_O_2_; <$>\raster(70%)="rg3"<$> H_2_O_2_+ salidroside (0.1µM); <$>\raster(70%)="rg4"<$> H_2_O_2_+ salidroside (1µM); <$>\raster(70%)="rg5"<$> H_2_O_2_+ salidroside (10µM); <$>\raster(70%)="rg6"<$> H_2_O_2_+NAC(1 mM). B: Effect of salidroside on the ALP activity of MC3T3-E1 cells in the presence of H_2_O_2_. The control value for ALP activity was 0.682 ± 0.021 unit /µg protein. C: Effect of salidroside on the mineralization of MC3T3-E1 cells in the presence of H_2_O_2_. The control value for mineralization was 1.392±0.31 OD. D: Effect of salidroside on the mRNA expression of *Alp*, *Col-1* and *Ocn* in the presence of H_2_O_2_. ^#^ *P*<0.05 versus untreated control cells;*P<0.05 and **P<0.01 compared with the group treated with H_2_O_2_ alone.

In order to assess the effect of salidroside on osteoblast dysfunction induced by H_2_O_2_, ALP staining and activity, calcium deposition and osteogenic differentiation genes (*Alp*, *Col1a1*, *Ocn*) were determined. Compared with the control cells, the presence of H_2_O_2_ significantly decreased cellular ALP activity, mineralization and expression of osteogenic differentiation genes (Fig.3). When osteoblasts were pretreated with salidroside in the presence of H_2_O_2_, salidroside (0.1-10µM) significantly increased cellular ALP activity, which is one of the major osteoblast differentiation markers (Fig. 3B). We further investigated the effect of salidroside on mineralization in the presence of H_2_O_2_ by measuring calcium deposition by Alizarin Red staining. As shown in Fig. 3C, salidroside (0.1–1µM) showed significant recovery effect on mineralization inhibited by H_2_O_2_. Moreover, salidroside supplementation significantly enhanced the expression of osteogenic differentiation genes (*Alp*, *Col1a1*, *Ocn*) compared with the group treated with H_2_O_2_ alone (Fig. 3D). Our results demonstrated that salidroside attenuates osteoblast dysfunction induced by H_2_O_2_.

### Inhibition of salidroside on RANKL and IL-6 production of MC3T3-E1 cells in the presence of H_2_O_2_

**Figure 4:**
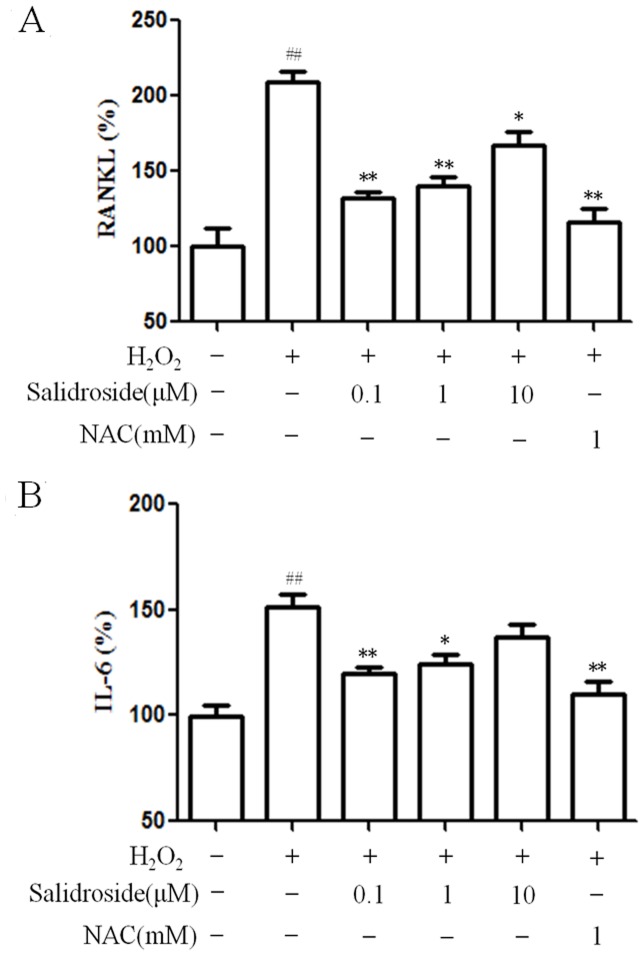
Inhibition of salidroside on RANKL and IL-6 production of MC3T3-E1 cells in the presence of H_2_O_2_. After differentiation induction of 6 days, MC3T3-E1 cells were pretreated with salidroside for 24 h before treatment with 0.3 mM H_2_O_2_ for 24 h. NAC was used as positive control. A: The production of RANKL in the presence of salidroside and/or H_2_O_2_. The control value for RANKL was 3.792±0.271ng/mg. B: The production of IL-6 in the presence of salidroside and/or H_2_O_2_. The control value for IL-6 was 0.429±0.391 ng/mg. ^##^ *P<*0.01 versus untreated control cells;*P<0.05 and **P<0.01 compared with the group treated with H_2_O_2_ alone.

In order to determine the possible regulation of osteoclast differentiation by osteoblasts, we examined the production of RANKL and IL-6, which are primarily released from osteoblast cells. Osteoblast-derived RANKL binds to RANK on osteoclasts, resulting in osteoclast activation, and IL-6 have also been demonstrated to increase osteoclastic activity. When 0.3 mM H_2_O_2_ was added to the cells, the production of RANKL and IL-6 increased significantly. However, H_2_O_2_-induced RANKL and IL-6 production was significantly inhibited by pretreatment of salidroside at concentrations of 0.1–10µM and 0.1–1µM, respectively (Fig.4).

### Salidroside reduces the production of ROS induced by H_2_O_2_

**Figure 5:**
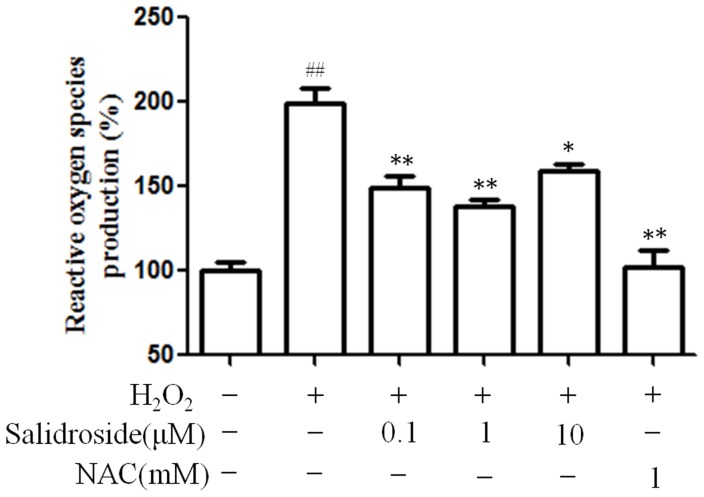
Inhibition of salidroside on reactive oxygen species generation induced by H_2_O_2_ in MC3T3-E1 cells. After differentiation induction of 6 days, MC3T3-E1 cells were pretreated with salidroside for 24 h before treatment with 0.3 mM H_2_O_2_ for 24 h. NAC was used as positive control. The data shows changes in levels of ROS, which was measured by DCF fluorescence method. ^##^ *P*<0.01 versus untreated control cells;*P<0.05 and **P<0.01 compared with the group treated with H_2_O_2_ alone.

To investigate whether cell-protective action of salidroside is related to its antioxidant activity, the production of ROS detected by fluorescent probe DCFH-DA was assessed. As shown in Fig. 5, the production of ROS was increased significantly by treatment of 0.3 mM H_2_O_2_. These results suggest that H_2_O_2_ enhanced oxidant generation and may cause damage to osteoblastic MC3T3-E1 cells. However, the production of ROS was significantly inhibited when pretreated with salidroside at concentrations of 0.1–10µM (Fig.5). These data support the hypothesis that the cytoprotective effect offered by salidroside may be associated with its antioxidant capacity.

### Inhibition of salidroside on oxidative status in serum

**Figure 6:**
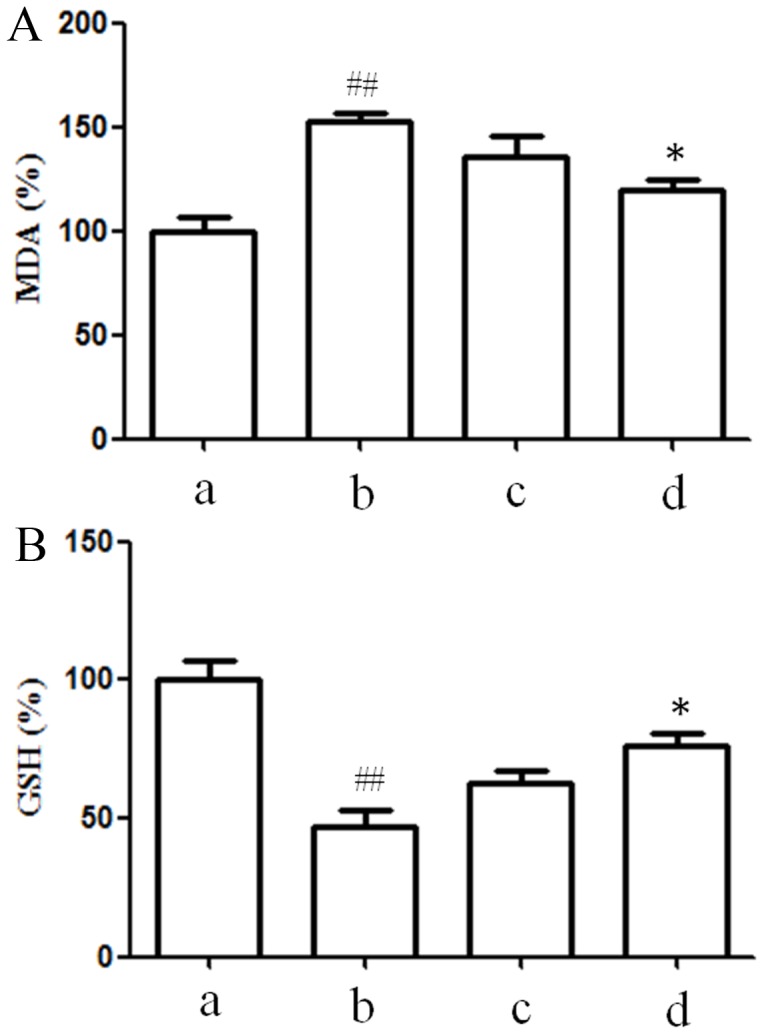
Inhibition of salidroside on oxidative status in serum of ovariectomized mice. A: Serum MDA concentrations in ovariectomized mice supplemented with different concentration of salidroside. B: Serum GSH concentrations in ovariectomized mice supplemented with different concentration of salidroside. a: sham; b: OVX; c: OVX+salidroside (5 mg/kg); d: OVX+salidroside (20 mg/kg). The control value for MDA and GSH was 7.692±0.928 nM/mg and 13.24±1.391 nM/mg.^##^ *P*<0.01 versus sham group;*P<0.05 compared with OVX group.

Serum MDA and GSH levels are potential biomarkers for measurement of oxidative stress. As shown in Fig. 6, serum MDA level was increased and GSH level was decreased in OVX mice when compared with sham groups (*P*<0.05). Salidroside supplementation (20 mg/kg) significantly decreased serum MDA concentrations and increased GSH level (*P*<0.05). No differences in MDA and GSH levels were observed in OVX mice treated with different concentrations of salidroside (*P*>0.05).

### Protective effect of salidroside against bone loss in OVX mice

**Figure 7:**
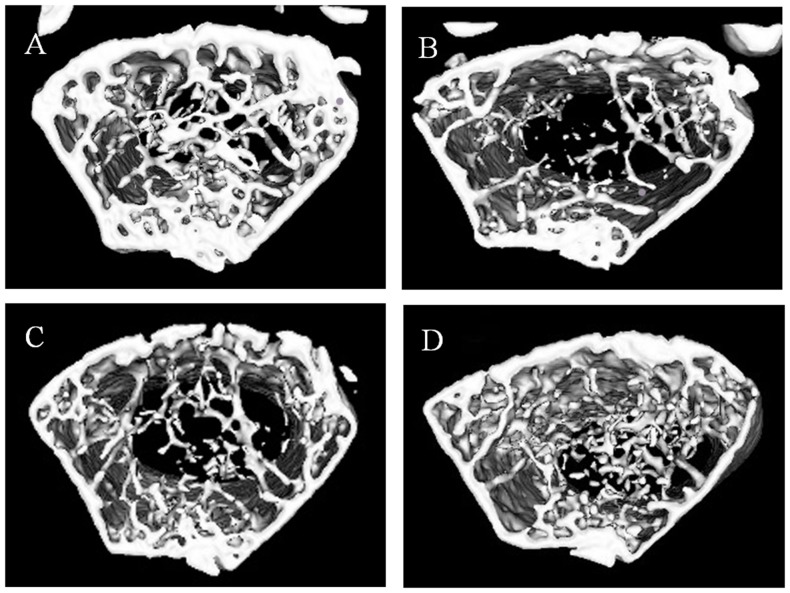
Analysis of micro-computed tomography within the distal metaphyseal femur region. A: sham, B: OVX, C: OVX+salidroside(5 mg/kg), D: OVX+salidroside(20 mg/kg).

**Figure 8:**
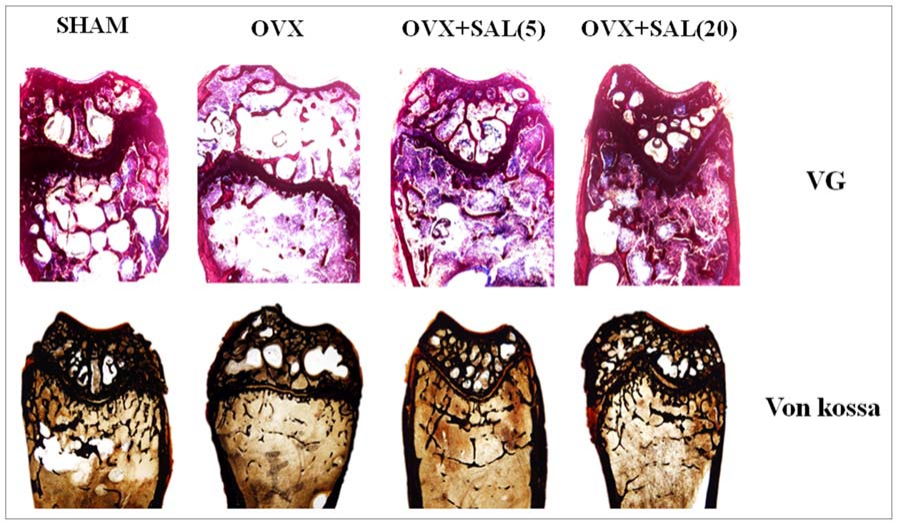
Van Gieson (VG) and Von Kossa (Silver nitrate) staining of the distal femur. The figure was 40× of the original section.

We administered salidroside as early as 7 days after ovariectomy to evaluate its effects on trabecular bone microarchitecture. We sacrificed all the animals 16 weeks after the operation. No significant difference in body weight of the mice in four groups was noted. The analysis of the properties of trabecular bone in distal femoral metaphyses and lumber vertebrate (L4) indicated that ovariectomy induced deterioration of the trabecular bone microarchitecture in mice, as demonstrated by the reduction in BV/TV, Conn.D, Tb.N, Tb.Th and BMD when compared with the sham group mice (*P*<0.01) (Table 2). In contrast, as shown in Table 2, SMI and Tb.Sp were significantly increased in response to OVX (*P<0.05* for both). However, treating OVX mice with a high dose of salidroside at 20 mg/kg could significantly (*P*<0.05) reverse the changes in these parameters induced by ovariectomy and could maintain the microarchitecture of trabecular bone in distal femoral metaphyses and lumber vertebrate (L4).These increments in trabecular bone parameters were readily observable in micro-CT images of the distal femur (Fig. 7). Salidroside at 5 mg/kg improved the trabecular bone mass loss and microarchitecture deterioration, but no significant difference was noticed (P>0.05) when compared with the OVX group. Van Gieson and Von Kossa staining also showed similar results (Fig. 8). Compared with the sham group, the trabecular number was reduced and spaces between trabecules were broader in the OVX groups. Salidroside supplementation inhibited these deleterious effects, as demonstrated by an increase in the trabecular number and a decrease in the trabecular space in the OVX+salidroside groups (Fig. 8).

**Table 2:** Effect of salidroside on bone microarchitecture indices in mice measured by Micro-CT.

Indices	Sham	OVX	OVX+salidroside(5)	OVX+salidroside(20)
**Distal femur**				
BV/TV (%)	0.107±0.029	0.031±0.004^##^	0.041±0.002*	0.073±0.011^**^
Tb.Th. (mm)	0.032±0.003	0.021±0.002^##^	0.025±0.002	0.028±0.004*
Tb.N. (1/mm)	3.59±0.331	1.441±0.111^##^	1.762±0.136	2.96±0.433^**^
Tb.Sp. (mm)	0.250±0.024	0.676±0.053^##^	0.579±0.048*	0.317±0.047^**^
Conn.D (1/mm^3^)	107.004±15.928	32.739±9.391^##^	45.551±7.697	66.516±10.429^**^
SMI	2.346±0.079	3.299±0.249^##^	2.925±0.187^**^	2.658±0.143^**^
BMD (mg/cm^3^)	213.043±22.29	120.028±14.94^##^	139.135±20.283	161.576±27.259^**^
**Lumbar vertebra**				
BV/TV (%)	0.189±0.562	0.093±0.031^##^	0.128±0.007^**^	0.145±0.081^**^
Tb.Th. (mm)	0.049±0.015	0.028±0.007^##^	0.036±0.011	0.043±0.009*
Tb.N. (1/mm)	4.753±1.427	2.897±0.678#	3.745±1.087	4.054±0.873
Tb.Sp. (mm)	0.184±0.065	0.365±0.083^##^	0.277±0.088	0.218±0.053*
Conn.D (1/mm^3^)	154.732±17.973	68.537±12.338^##^	85.447±13.46	119.781±15.749^**^
SMI	1.649±0.183	2.524±0.12^##^	2.171±0.21^**^	1.867±0.159^**^
BMD (mg/cm^3^)	183.984±20.14	92.661±15.592^##^	121.059±26.827	143.759±22.349*

Data were expressed as means ± S.D., n = 8 in each group.

# *P*<0.05 and ^##^ *P*<0.01vs. the sham group.

* *P*<0.05 and ^**^ *P*<0.01vs. the OVX group.

## Discussion

Oxidative stress is characterized by an increased level of reactive oxygen species (ROS) that disrupts the intracellular reduction–oxidation (redox) balance. Numerous studies have suggested that increased oxidative stress is involved in the pathogenesis of osteoporosis, caused by aging and estrogen deficiency [23], [24]. H_2_O_2_ is generated from nearly all sources of oxidative stress and has been demonstrated to play a crucial role in estrogen deficiency osteoporosis [25]. Therefore, we used H_2_O_2_-induced oxidative stress model in the present study. Fatokun reported that exposure of MC3T3-E1 cells to a concentration of at least 0.2 mM H_2_O_2_ for 1 h was required to produce significant lethality involving both apoptosis and necrosis [26].Our results showed that H_2_O_2_ substantially reduced cell viability in a dose-dependent manner, but the toxic effect was reversed to some degree when pretreated with different concentrations of salidroside for 24 h. Our previous studies revealed that salidroside protects against (MPP+)-induced apoptosis in PC12 cells, it can provide neuroprotective effects against focal cerebral ischemia in vivo and H_2_O_2_-induced neurotoxicity in vitro [17], [18]. The present study indicated that salidroside reduced the cytotoxicity induced by H_2_O_2_ in osteoblastic MC3T3-E1 cells.

Apart from affecting cell viability, oxidative stress also influences osteoblast differentiation. Liu et al. showed that H_2_O_2_ induced oxidative stress and suppressed the osteoblastic differentiation in primary mouse BMSCs [27]. This inhibition of cell differentiation was characterized by a reduction in ALP activity, which is an early differentiation marker and is important to regulate mineralization of bone matrix [28]. Subsequently, Bai et al. reported that H_2_O_2_-treated osteoblasts exhibited a reduction in levels of differentiation markers including *Alp*, *Co1a1*, colony-forming unit-osteoprogenitor (CFU-O) formation, and nuclear phosphorylation of the transcription factor *Runx2* [29]. More recently, it was indicated that H_2_O_2_ diminished mineralization and decreased the expression of osteogenic genes *Runx2*, *Alp* and bone sialoprotein in MC3T3-E1cells [30]. The present study has demonstrated that H_2_O_2_ significantly decreased cellular ALP activity, mineralization and expression of osteogenic differentiation genes *Alp*, *Col1a1* and *Ocn*, which confirms the previous observations that H_2_O_2_ toxicity leads to dysfunction of osteoblasts. Meanwhile, we found that the inhibition induced by H_2_O_2_ of osteogenic differentiation can be reversed by salidroside. According to our unpublished date, salidroside alone could not improve the proliferation and osteogenic differentiation of MC3T3-E1 cells significantly within the observed concentration. Therefore, we deduced that the protection of salidroside may mainly contribute to its antioxidant ability, suggesting that salidroside may be a beneficial agent in preventing osteoporosis associated with oxidative stress by enhancing osteoblast function.

Osteoblasts are coupled with osteoclasts in terms of the release of various bone-resorbing cytokines, such as receptor activator of nuclear factor (NFκB) ligand (RANKL) and IL-6. RANKL, highly expressed on osteoblasts, marrow stromal cells and T cells, is an essential cytokine involved in osteoclastogenesis. RANKL acts by binding to the RANK receptor on osteoclast progenitor cells, leading to expression of osteoclast differentiation genes, prolonged survival of osteoclasts and increased bone resorption [31]. RANKL is prevented by osteoprotegerin (OPG), a soluble decoy receptor that competes with RANK for binding to RANKL and is also expressed by osteoblasts [32]. Bai et al. reported that ROS stimulates RANKL expression in mouse osteoblasts and human MG63 cells [33]. IL-6 was also reported to be produced by osteoblasts [34] and can induce RANKL mRNA expression, promote the differentiation of osteoclasts from its precursor and play an important role in the pathogenesis of osteoporosis due to estrogen deficiency [35], [36]. ROS might indirectly stimulate osteoclasts by augmenting expression of resorptive cytokines such as RANKL, TNF-α and IL-6 that have been strongly implicated in estrogen deficiency bone loss [37]. In this study, salidroside inhibited the production of RANKL and IL-6 induced by H_2_O_2_ in osteoblastic cells. The inhibitory effect on RANKL and IL-6 production may contribute to bone anti-resorbing effect of salidroside, and may play a role in the reduction of bone loss.

Reactive oxygen species (ROS) such as superoxides anions, hydroxyl radicals, and H_2_O_2_ can cause severe damage to DNA, protein, and lipids [38]. High levels of oxidant produced during normal cellular metabolism or from environmental stimuli perturb the normal redox balance and shift cells into a state of oxidative stress. Accumulating evidence suggests that ROS may play its role in bone loss-related diseases in two ways: suppression of bone formation and stimulation of bone resorption. Salidroside is extracted from Rhodiola rosea L, which is one of Chinese traditional medicine, has been reported to possess antioxidative properties [14]. In the present study, pretreatment with salidroside for 24 h could reverse the production of ROS induced by H_2_O_2_ to some degree. Therefore, the results of the present study demonstrated that salidroside can act as a biological antioxidant and protect cells from oxidative stress-induced toxicity. Accordingly, the protective effect provided by salidroside to osteoblastic MC3T3-E1 cells might be mediated, at least in part, via its antioxidant ability.

Protection against oxidative stress is a possible mechanism explaining salidroside's beneficial effects. However, there is a paucity of knowledge regarding its molecular mode of action. We speculated that it may degrade H_2_O_2_ directly, elevate the endogenous antioxidant defenses or through ROS-irrelevant mechanisms. Wiegant et al. reported that Rhodiola rosea L. could increase expression of hemeoxygenase-1 (HO-1), a protein that can be activated by the antioxidant-response element (ARE) in response to oxidative challenge [39]. ARE is a *cis*-acting enhancer element in the 5′flanking region of the cytoprotective enzymes. This element regulates many antioxidant enzymes including the glutathione *S*-transferases, HO-1 and NQO1, and its activation by transcription factor nuclear erythroid-2 related factor-2 (Nrf-2) confers a resistance to oxidative damage. Salidroside is an active constituent extracted from Rhodiola rosea L. Therefore, we speculate that salidroside provided protection effects against H_2_O_2_-induced cytotoxicity and osteoblast dysfunction in MC3T3-E1 cells may through the HO-1 and Nrf-2 signaling pathways. We will confirm this molecular mechanism in the further study.

The mechanisms through which estrogen deficiency stimulates bone loss remain controversial. Recently, oxidative stress has been suggested to be responsible for the development of postmenopausal osteoporosis. Serum MDA levels is one of the potential biomarkers for oxidative stress [40]. Lipid peroxides (MDA) is the end product of lipid peroxidation caused by ROS. It can cause increased cell membrane permeability [41]. Reduced glutathione is one of the most important reducer agents which maintain the intracellular redox balance [42]. Decrease of GSH level is also a marker for oxidative stress [43]. In our study, salidroside treatment for 3 months prevented the increased serum MDA level and decreased GSH level resulting from OVX. Our results indicated that salidroside could improve the oxidative status in estrogen deficient osteoporosis model. Bone quality is determined by microarchitecture, geometry and material properties of the bone. Measuring such microarchitectural parameters as BV/TV, Tb.N, Tb.Sp, Tb.Th, Conn.D, SMI and BMD may improve our ability to estimate bone strength [44], [45]. The effects of ovariectomy on bone are smaller in cortical compartments than in trabecular compartments [46].Therefore, we observed the effect of salidroside on trabecular microarchitecture by scanning with Micro-CT. All parameters, such as BMD, BV/TV, Tb.N, and Tb.Sp, showed that salidroside treatment with 20 mg/kg for 3 months improved bone structural indices, bone mineral density and trabecular thickness compromised by OVX. Meanwhile, histological examination also showed that ovariectomy had negative effects on the microarchitecture of the trabecular bone. However, this effect could be reversed by supplementation of salidroside to some degree. These data implied that salidroside may significantly increase trabecular bone mass in ovariectomized mouse model and salidroside has a therapeutic effect in prevention of estrogen-deficient osteoporosis.

In conclusion, our studies demonstrated that salidroside protects MC3T3-E1 cells from H_2_O_2_-induced cell damage and dysfunction of osteoblasts via inhibition of oxidative stress and bone-resorbing mediators. Moreover, salidroside reduces the oxidative status in serum and prevents bone loss and deterioration of trabecular microarchitecture in OVX-induced osteoporosis mouse model. Our results suggest that salidroside could be used as a good candidate for preventing and treating osteoporosis. Further investigation is required in order to clarify the detailed molecular mechanisms of action of salidroside in bone.
